# Volatile outcomes of essential public health functions: a cross-sectional study of surveillance and equitable access on Brazil’s Unified Health System (SUS)

**DOI:** 10.3389/fpubh.2025.1613822

**Published:** 2025-06-11

**Authors:** Alessandro Jatobá, Paula Castro-Nunes, Paulo Victor Rodrigues de Carvalho

**Affiliations:** Oswaldo Cruz Foundation (Fiocruz), Rio de Janeiro, Brazil

**Keywords:** health systems resilience, public health, health services accessibility, health equity, surveillance

## Abstract

**Background:**

This study investigates the volatility in the outcomes of Essential Public Health Functions (EPHFs) and elaborates on its potential impacts on the operation of Brazil’s Unified Health System (SUS). The research addresses the need to understand how performance variability in EPHFs affects health system stability, particularly during external shocks such as the COVID-19 pandemic, and its potential effects on the system’s resilience.

**Methods:**

Using cross-sectional data (2000–2023) from the Department of Informatics of the SUS (DATASUS), the study analyzes key indicators linked to two EPHFs: (1) Surveillance, control, and risk management (infant mortality) and (2) Equitable access to comprehensive and quality services (cytopathological tests and mammography screenings). Volatility was defined as deviations from central trends exceeding one standard deviation relative to prior years. These metrics were assessed to evaluate health system performance and resilience.

**Results:**

Significant volatility was observed across indicators, particularly during the COVID-19 pandemic, which disrupted service levels and exposed systemic vulnerabilities. Infant mortality declined by 60% since 2000 but exhibited persistent fluctuations. Cytopathological tests and mammography screenings saw sharp declines during the pandemic, reflecting systemic challenges in sustaining equitable access to care.

**Conclusion:**

The study proposes a conceptual framework to analyze EPHF performance through a resilience lens, emphasizing the need to manage variability for stable, high-quality service delivery in the SUS. Recommendations include strengthening health data systems, integrating contextual factors into resilience planning, and enhancing institutional capacity. This work advances efforts to operationalize resilience assessments in universal health systems, offering actionable insights for policymakers and practitioners.

## 1 Introduction

This article explores the performance of the outcomes of essential public health functions (EPHF) of the Brazil’s Universal Health System (SUS), one of the largest health systems centered on universal access (1), with the objective of describing the EPHF’s outcomes’ volatility trough time.

Charles Winslow proposed an inventory of EPHFs at the beginning of the 20th century (2). Although there was some consensus on Winslow’s proposals, the practical limitations in government, the private sector, and society have evolved greatly over time. For example, until the middle of the last century, the functions of public health were limited to sanitation, basic hygiene, and the management of communicable diseases. Over time, the field has gradually broadened its scope to include health promotion, the control of non-communicable diseases, access to primary care, and technology applied to the various areas of health.

The World Health Organization (WHO) published its first inventory of EPHFs in 1997 and updated it after 15 years (3). Since the late 1990s, four WHO regions – Europe, the Western Pacific, the Americas and the Eastern Mediterranean – have developed their own lists of EPHFs. However, recent events, such as the H1N1 flu in 2009, the Ebola outbreak in West Africa in 2014, and the Zika virus in the Americas in 2016, in addition to the Covid-19 pandemic, have once again brought the need to review inventories of EPHFs, in particular to make health systems more resilient, toward achieving universal coverage aligned with the Sustainable Development Goals (SDGs) listed in the 2030 Agenda (4).

To support the Member States in developing comprehensive plans and policies for the health sector, the Pan American Health Organization (PAHO) published in 2020 a new inventory of EPHFs in the Americas, focused on resilience and more aligned with the SDGs and the principles of Universal Access to Health and Universal Health Coverage promoted by the WHO (5).

The document published by the PAHO is quite detailed regarding the recommended operation of the functions, which are supported by three pillars: introducing ethical values in public health action to address health inequalities and their root causes; ensuring universal access to comprehensive public health services, both individual and population-based; expanding the administrative role of health authorities through a collaborative implementation of public health functions.

Although the EPHFs aim to sustain the quality of life of populations, their practical use depends on how a given health system configures its institutional capacity dimensions within reasonable and possible limits. Health systems are constantly affected by external factors such as epidemics, climate change, or internal factors as changes in governance arrangements or are unable to mobilize funding, human resources or technology at acceptable levels (6). Operating in such context, health systems struggle to sustain adequate functioning, readjusting constantly. Regardless of the level at which variability occurs – from governance down to service delivery – it leads to volatility in known performance indicators over time, affecting the precision and/or timing of systems’ capacities, service levels, availability, and quality of care.

The functioning of public and universal health systems depends on structural dimensions that guide EPHFs in various directions. Disturbances in these dimensions inevitably alter the outcomes of these functions. Such maneuvers, referred to as performance variability in the field of Resilience Engineering (7) may resonate throughout the system’s functioning and lead to uncontrolled or chaotic system behavior, eventually leading to critical failure, according to Dekker (8). Therefore, by enhancing our understanding of EPHF dynamics, it is possible to deliver new insights to public health officials, helping them to better manage system capacities for resilience. Ultimately, this will save lives and build a more resilient SUS.

The aim of this study is to connect the variability in the EPHFs’ performance to an objective assessment of SUS capacity to sustain responsive EPHF. The rationale is that a resilient health system should be able to adjust its functions to generate stable functions’ outcomes within acceptable health services levels. This means that, even if necessary adaptations involve performance variability, resilient systems adjust their functions internally to provide stable and adequate outcomes, sustaining responsive service delivery and acceptable quality of care.

## 2 Methods

### 2.1 Research design

This cross-sectional study was based on public data from the Department of Informatics of the Brazilian Unified Health System (DATASUS). The study was conducted in accordance with the recommendations of the Strengthening the Reporting of Observational Studies in Epidemiology (STROBE) checklist.

To illustrate the framework’s idea, we selected two key EPHFS, guided by convenience: “Surveillance, control, and risk management”; and “Equitable access to complete and quality services.” These functions were chosen because they are crucial performance indicators and susceptible to disruption based on their connections with other functions. Brazil was selected as our study site because it’s where our research group operates and its health system data is easily accessible. Additionally, Brazil’s health system aligns with principles of universal and equitable access.

### 2.2 Research settings

Since 1988, with the publication of Brazil’s current Constitution, the Unified Health System (SUS) has become one of the largest and most complex public health systems in the world. Among countries with more than 200 million inhabitants, Brazil is the only publicly funded universal health system. The DATASUS (Department of Informatics of the Unified Health System - SUS) is a unit of the Brazilian Ministry of Health that has been developing and managing Brazil’s health information systems since 1991.

Epidemiological indicators in Brazil have improved over time, especially those related to quality of life. However, due to an incomplete demographic-epidemiological transition and the prevalence of the triple burden of disease, many challenges toward comprehensive, equal, and universal access remain (9, 10). Such a complex scenario necessitates robust public health strategies to address both emerging and persistent health issues.

The analysis of the EPHFs “Surveillance, control, and risk management” and “Equitable access to complete and quality services” is particularly justified in this context. Effective surveillance and risk management are critical for monitoring disease patterns, identifying health threats, and implementing timely interventions, especially in a country grappling with diverse health challenges. Simultaneously, ensuring equitable access to comprehensive and high-quality health services is essential to address disparities and improve health outcomes, particularly for vulnerable populations disproportionately affected by the triple burden.

### 2.3 Data collection procedure

The dataset was built with indicators for all Brazilian states from 2000 to 2023. The indicator “infant deaths from preventable causes in children under 5 years of age” was defined as the outcome variable of the model, as well as “Cytopathological tests” and “Mammography exams.” Such outcomes do not encompass the entire set of outcomes related to the chosen EPHFs, but they serve to exemplify the proposed framework as such numbers indicate the level at which people from vulnerable territories are accessing public health services.

The data were collected from the following information databases of the Department of Informatics of the SUS (DATASUS): the Mortality Information System (SIM); the Epidemiological and Morbidity – Cancer Information System (SISCAN); and the Breast Cancer Information System (SISMAMA).

### 2.4 Data analysis procedure

The numbers of cytopathological tests and mammograms are outcomes of the “Equitable access to complete and quality services “EPHF, according to the PAHO (5). The number of infant deaths is one of the outcomes of the “Surveillance, control, and risk management” function. Measurements that diverge from the central trends based on the values of previous years in dimensions greater than the standard deviation were considered volatile. This observation is also used to estimate the magnitude of the trend change, which is important for epidemiological and economic analyses. In the temporal trends analysis, a significant level of 5% was established.

All analyses were performed with the support of a routine in the statistical *software* R Studio version 2023.06.0.

## 3 Results

**Figure 1:**
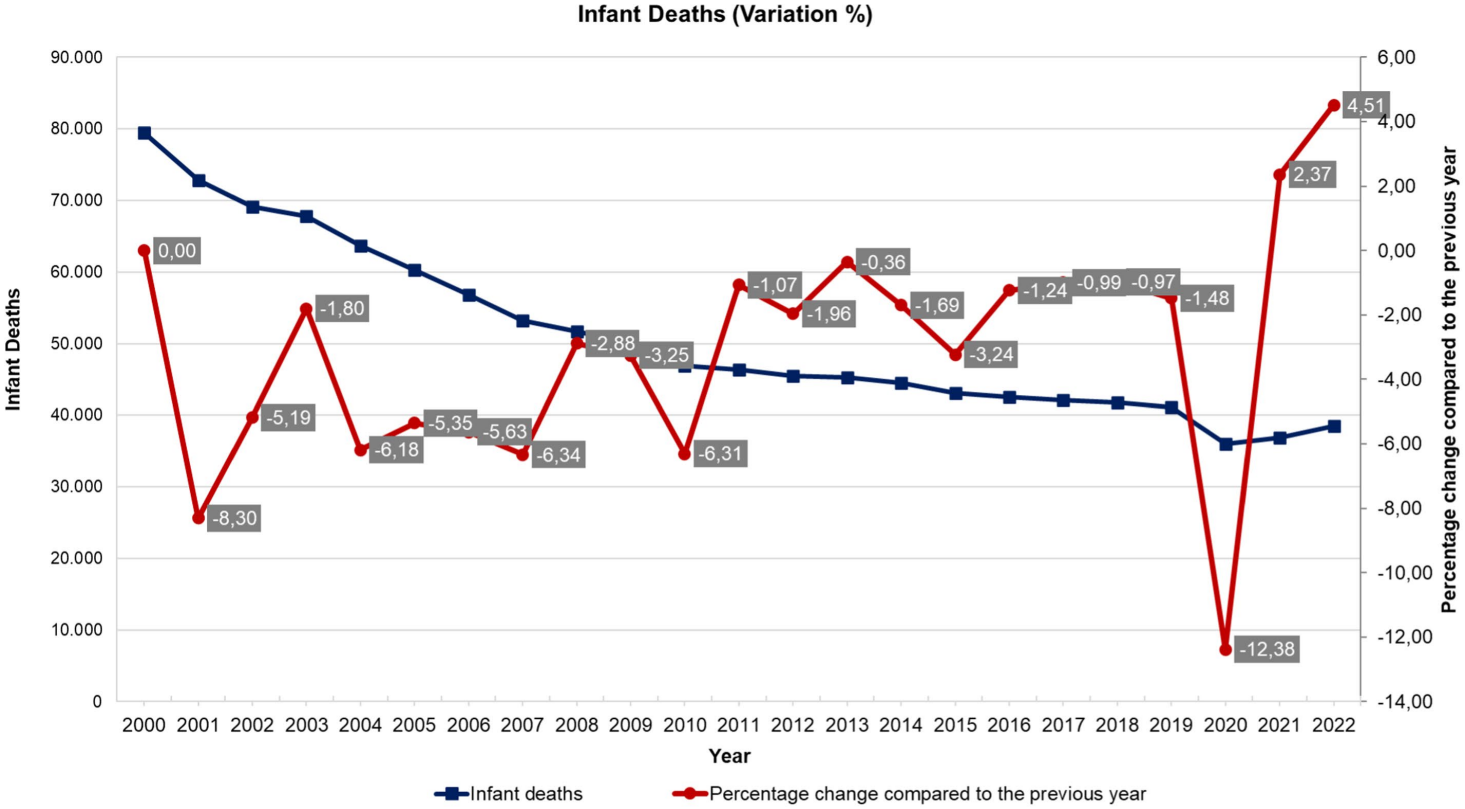
Historical series of volatility (percentage change) of the number of infant deaths in Brazil - national average (2001 to 2022). Source: Department of Informatics of the Unified Health System (DATASUS), Ministry of Health, Brazil.

Figure 1 represents the evolution of the number of infant deaths from 2001 to 2023.

It is worth noting that although infant mortality has decreased by 60% since 2000, there is great volatility in the still high levels of mortality, which reflects the lack of continuity in public social and health policies related to the quality of services to improve the quality of life of the population, such as primary health care workforce, vaccination coverage, health unit infrastructure, prenatal, childbirth and newborn monitoring and care (nutrition, breastfeeding, proper growth and development), surveillance and control of communicable diseases (such as Zika and cholera), basic sanitation, etc.

**Figure 2:**
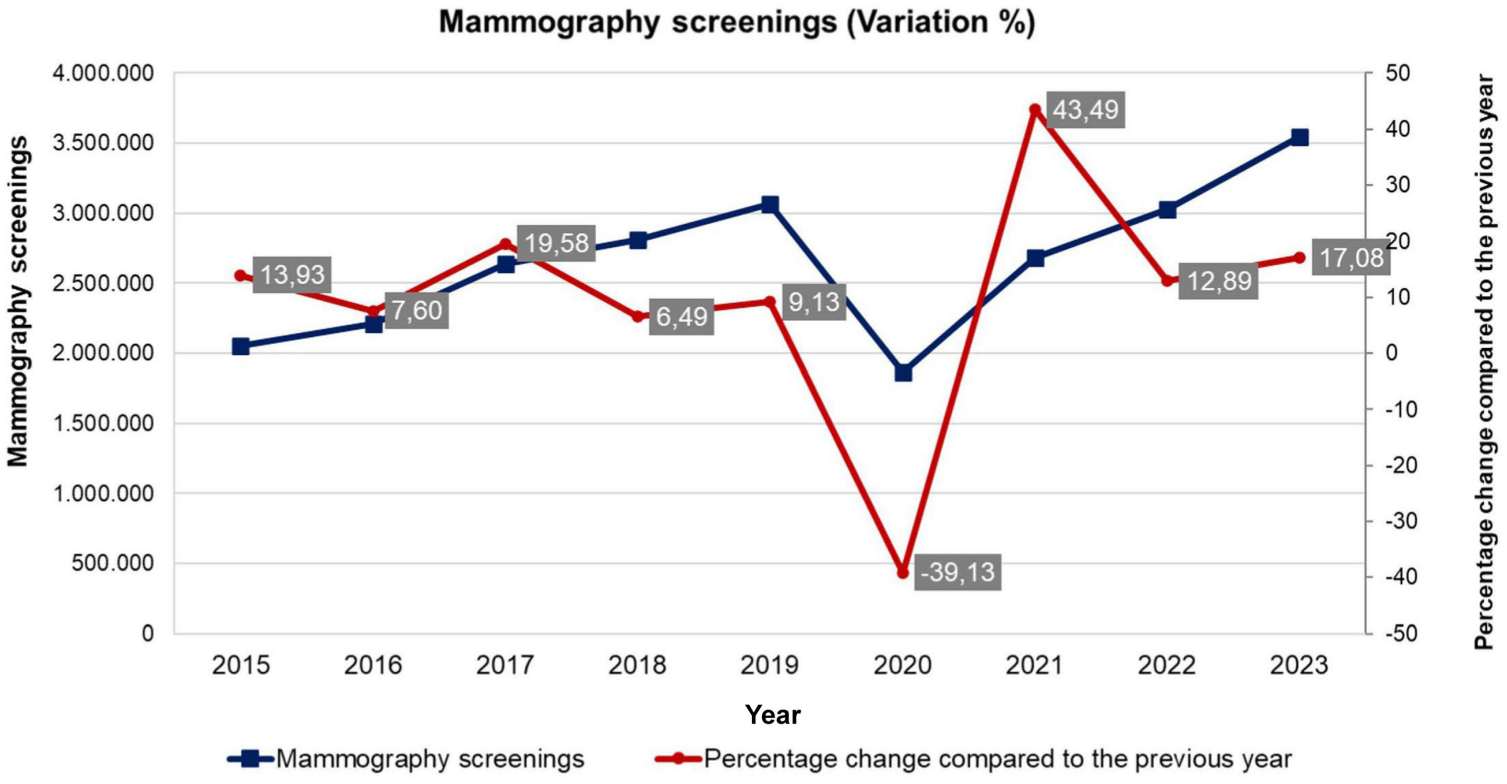
Historical series of volatility (percentage change) of the number of mammograms performed in Brazil - absolute numbers (2015 to 2023). Source: Department of Informatics of the Unified Health System (DATASUS), Ministry of Health, Brazil.

The “Equitable access to comprehensive, quality health services” function includes actions to ensure access to comprehensive and quality services, progressively expanded, through the organization and management of patient-centered health services, focused on family and community risk, life course, and social determinants of health. Health system utilization indicators result from this function, such as the number of cytopathological tests, as shown in Figure 2.

**Figure 3:**
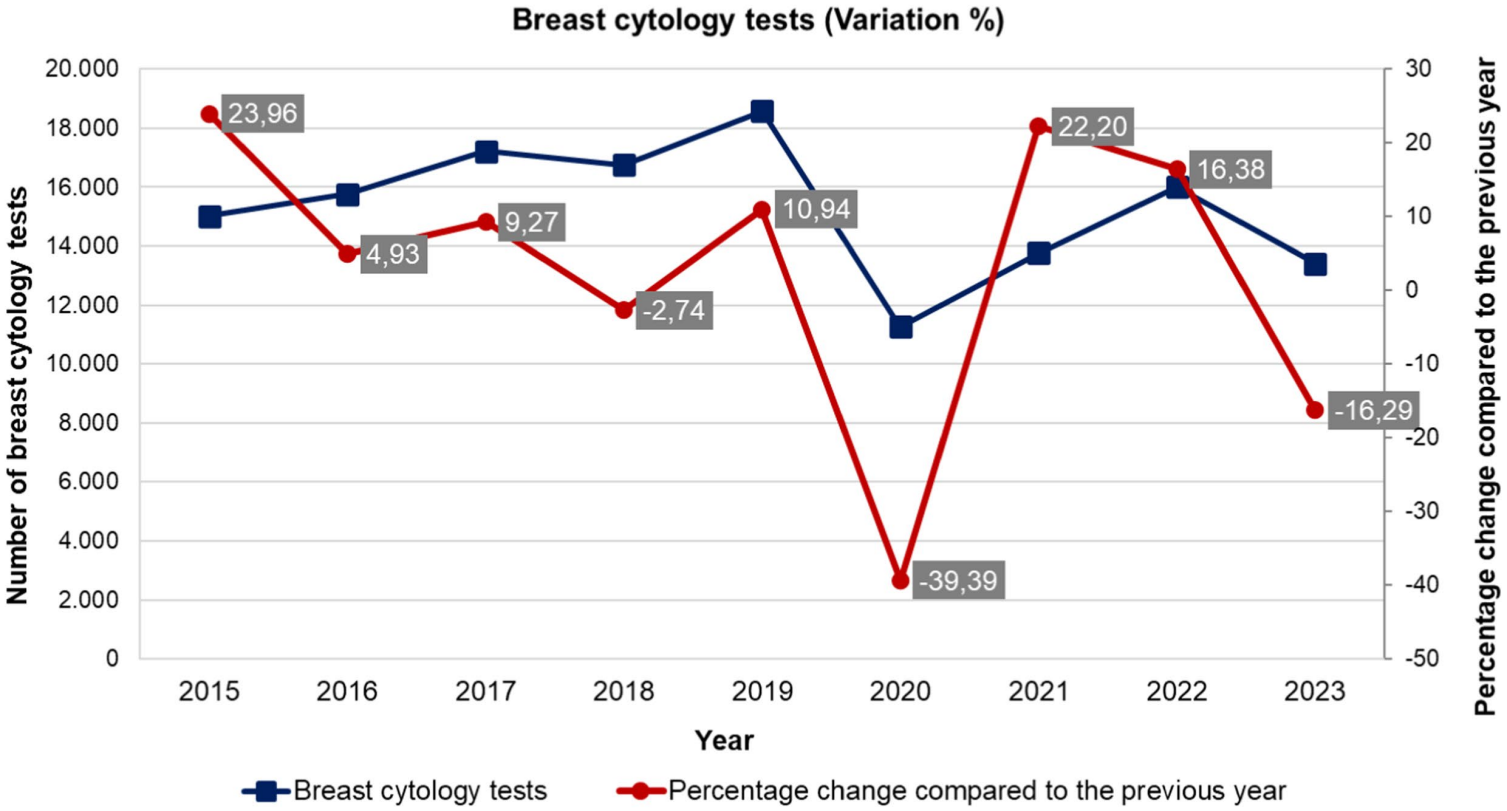
Historical series of volatility (percentage change) of the number of breast cytopathology tests performed in Brazil - absolute numbers (2013 to 2023). Source: Department of Informatics of the Unified Health System (DATASUS), Ministry of Health, Brazil.

Figure 3 describes the volatility of exams performed in Brazil between 2013 and 2023. It should be noted that the curve of the number of tests performed in the period had been on an upward trend until 2019, when it fluctuated, but has not yet returned to pre-COVID-19 levels. This volatility demonstrates the effects of the lack of continuity of health services, delaying the diagnosis of breast cancer.

Still on the provision of services and performance of the public health system, it is noted in Figure 2 that the high volatility of mammograms impairs the correct and continuous provision of health services, which has a decisive impact on access, affecting the resilience of health systems. It is noteworthy that the obstacles to the diagnosis of breast cancer (cancer with the highest lethality rates among women in the world, except for non-melanoma skin cancer), may mean the reduction of opportunities for clinical and surgical feasibility, expanding the implementation of the more mutilating and aggressive procedures or palliative care.

## 4 Discussion

Traditional epidemiological frameworks, like multifactorial disease models, have long emphasized the inherent complexity of public health challenges (11–13). These approaches aggregate data across risk factors to inform intervention strategies. However, causal inferences derived from such frameworks often lack robustness, or generalizability to meet the inherent variability of such complexity (11, 13). Their retrospective focus on historical associations may limit assessments of resilience against novel threats, as they prioritize past events to generate recommendations that, while evidence-based, risk inadequacy in emerging or evolving contexts (14).

Resilient performance in Essential Public Health Functions (EPHFs) hinges on linking policy measures to measurable outcomes. Castro et al. (15) operationalized this by developing indicators to correlate COVID-19 transmission trajectories (e.g., speed, intensity) with policy interventions in Brazil. Their analysis revealed that the rapid spread stemmed not from a single cause but from fragmented implementation of timely, equitable, and coordinated responses. Building on this, Rocha et al. (16) demonstrated that systemic resilience extended beyond the capacity of Brazil’s SUS, as preexisting socioeconomic inequities—not healthcare infrastructure alone—drove disparities in COVID-19 morbidity and mortality, disproportionately burdening vulnerable states and municipalities. While these studies clarify how variability in policies and social conditions produces unstable EPHF outcomes, their focus remains on acute, episodic crises. However, systems like the SUS operate under chronic instability shaped by persistent regional disparities, political volatility, and resource constraints. True resilience requires sustaining stable EPHF outcomes amid shocks of varying intensity, frequency, and origin, as prolonged instability itself can amplify systemic collapse (17).

Figure 1 illustrates the trend of infant deaths from 2000 to 2022, showing both the number of deaths and the percentage change compared to the previous year. Initially, there is notable volatility in the percentage changes, particularly between 2000 and 2006, with significant fluctuations such as increases of 5.10% in 2001 and 6.69% in 2005. This period of instability could indicate underlying systemic issues or varying external factors affecting infant mortality. Connecting these results with Dekker’s concept of complex systems, constant volatility can cause systems to “drift into failure,” suggesting that the early years’ instability might have posed risks to the overall effectiveness of healthcare systems aimed at reducing infant mortality. However, from 2007 onwards, the trend stabilizes, with more consistent negative percentage changes indicating a steady decline in infant deaths. This stabilization aligns with improvements in healthcare practices, better prenatal care (18, 19), and enhanced public health interventions, reducing the risk of systemic failure and contributing to a more reliable and effective reduction in infant mortality over time.

Rocha et al.’s finding that socioeconomic inequities drove COVID-19 outcomes in Brazil mirrors the Castro et al.’s (1) argument that systemic brittleness in Latin America stems from entrenched inequalities. Our data on infant mortality trends (Figure 1) and SUS’s chronic instability further validate this: regions with preexisting vulnerabilities (e.g., low socioeconomic status) exhibited amplified volatility during crises. This aligns with their call for resilience frameworks to address structural determinants—not just proximate risk factors—to mitigate inequitable strain on EPHFs.

Resilience in public health is the result of the design, implementation, and maintenance of national systems capable of ensuring the uninterrupted, timely, problem-solving, and quality functioning of EPHFs during sudden fluctuations in demand (17, 20–22). This involves adjusting to the variability inherent in the implementation of public policies compatible with the social determinants of health, as well as providing universal, comprehensive, and equitable programs and services. Therefore, if health systems resilience is not only to cope with health disasters, but and also an issue to be considered daily, high volatility along time in EPHFs must be monitored and managed (20).

In Figure 3, the significant drop in breast cytology tests during the COVID-19 pandemic can indeed be related to the preceding volatility observed in the data. Prior to the pandemic, the graph shows fluctuating percentage changes in the number of tests conducted, with notable variations such as a 39.89% increase followed by a − 10.29% decrease. This volatility indicates an underlying instability in the system responsible for conducting these tests, and aligns with the dynamic, non-linear interactions described by Castro et al. (15), to whom systemic resilience arises from adaptive behaviors rather than static infrastructure. Like their analysis of pandemic responses, our findings suggest that pre-existing instabilities (e.g., fluctuating screening rates) reflect a system’s latent capacity to adapt—or fail—when confronted with shocks. This underscores the need to model resilience as an emergent property, as static metrics of institutional capacity alone cannot predict how systems will perform under stress. The pre-pandemic fluctuations suggest that the system was already under stress or lacked the robustness needed to handle sudden, large-scale disruptions (23, 24). When the pandemic hit, the existing instability likely exacerbated the system’s inability to maintain normal operations, leading to the sharp decline in test numbers.

In essence, the preceding volatility weakened the system’s resilience, making it less capable of sustaining critical functions like breast cytology tests during the unprecedented challenges posed by the pandemic. This highlights the importance of continually addressing underlying instabilities in health systems to enhance their ability to withstand future crises. The topic of resilience - or resilient performance - stresses the management models traditionally used to measure and evaluate the performance of health systems, since these models are usually based on the analysis of institutional capacity and infrastructure.

The mammography screenings from 2015 to 2021 analyzed in the Figure 2 reveals significant volatility and a notable decline during the COVID-19 pandemic. Initially, there is a sharp increase in 2015 with a 43.49% change, followed by fluctuating rates in subsequent years, indicating underlying instability in the system. This volatility suggests challenges in maintaining consistent screening rates, possibly due to varying public health initiatives or policy changes (6, 25). The pandemic in 2020 exacerbated these issues, causing a sharp drop to a 6.49% change, reflecting disrupted healthcare services and reduced patient access. Although there was a slight recovery in 2021 with a 9.13% change, the numbers remained below pre-pandemic levels. This pattern underscores the vulnerability of healthcare systems to crises when pre-existing instabilities are present, highlighting the need for more resilient and stable healthcare infrastructures to ensure continuous delivery of essential services like mammography screenings.

### 4.1 Institutional capacity and performance variability

Recent research indicates that while institutional capacity is necessary, it is not sufficient for the proper functioning of EPHFs in scenarios of abrupt change (17, 26, 27). Therefore, it is not a good predictor of how services adapt to unexpected events, maintain their essential functions during a crisis, nor how they learn from the experience and positively transform as they recover from shocks (26, 28–31). To address this, novel metrics and methods capable of describing the preventive, adaptive, and absorptive capacities of health systems are needed (27).

In 2022, the WHO took significant steps toward operationalizing the resilience of health systems by providing high-level definitions and offering a toolkit for implementing actions to strengthen health system resilience (32). A major advancement in the WHO’s conceptualization of resilience in this publication is the recognition of the need for multidisciplinary approaches and combined methodologies to develop resilience as an attribute of health systems. Additionally, there is an indication to consider health systems beyond their institutional capacity, focusing on how this capacity is operationalized through a set of skills, including perception, transformation, mobilization, self-regulation, integration, and diversity. Therefore, this attempt to link EPHFs’ outcome’ volatility to resilience enables a screening of institutional capacities ‘based on available data that guides further investigations on systems functioning, aimed at damping eventual volatility, especially if service levels are low.

**Figure 4:**
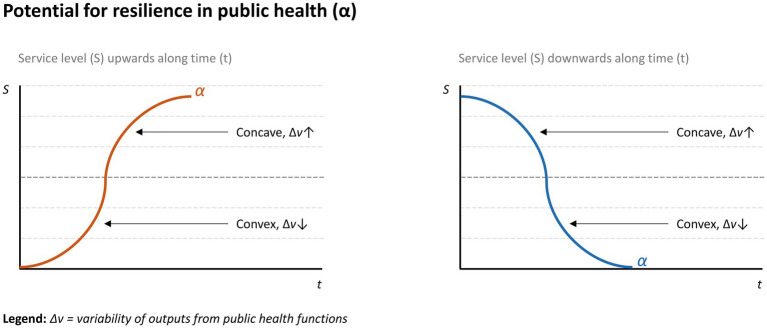
Graphical representation of resilience in public health. Potential resilient performance represented by curve α.

It is also important to highlight that, at this moment, the WHO reinforces an aspect usually forgotten in the conceptualization of resilience used by health authorities recently: that some functions considered essential for the health and well-being of populations must be preserved from crises that directly or indirectly affect people’s health. This definitively connects the building blocks of resilient systems (33, 34) to the EPHFs (5, 35) and the SDGs (4). The WHO’s recent emphasis on multidisciplinary resilience toolkits resonates with Bigoni et al. (36), which advocates for metrics that capture adaptive capacity in dynamic systems. Our proposed model (Figure 4), which correlates service levels (S) and outcome volatility (Δv), operationalizes this by quantifying how systems dampen variability while maintaining quality. This complements their framework, as both approaches prioritize real-time monitoring of instability (e.g., fluctuating mammography rates) to preemptively identify brittleness.

However, the WHO approaches are still tied to the notion that resilience should be an attribute activated only when a disaster strikes. Although it focuses radically on the behavior of complex systems, leaving aside the importance of the structure provided by institutional capacity, Resilience Engineering was already a counterpoint to this notion, highlighting resilience as an attribute to be developed, improved, and activated before, during, and after an unexpected event (7). This idea is especially useful to public and universal health systems, given their well-known complexity and susceptibility to variability, which materializes not only when major health disasters occur, but also routinely.

### 4.2 A theory for studying EPHFs’ performance variability, volatile outcomes, and resilience

Service levels result from the combination of different capacities and skills, employed in different components of a health system, and naturally vary over time, depending on resources, preconditions, capacity x demand proportion, etc. Thus, resilience in public health must also ensure adequate service levels, i.e., public health macro-functions must function stably with low levels of outcome volatility, but with high quality. There is no point in sustaining low volatility in the outcomes of EPHFs if the service levels do not guarantee responsive and comprehensive services.

Based on this, the potential for resilience in public health can be represented by the curve α arranged in a two-dimensional space that correlates service level (*S*) and time (*t*), whose concavity varies as a function of the volatility of the outcomes of the functions (Dv). Figure 4 illustrates these scenarios of both increase and decrease in the service levels over time.

The curvature of α is accentuated proportionally to the variability Dv, therefore, at low service levels, positive variabilities should be reinforced, which increase the convexity of α. Similarly, at high service levels, one should try to keep variability in stable proportions over time. In public health, the expectation of eliminating variability is not reasonable, given the influence of several dimensions on the functioning of the system. It is important, however, to manage variability at appropriate levels of services by operationalizing a resilient system as defined in this section – capable of sustaining the routine of EPHFs at appropriate levels of services and of adapting to fluctuations in demand, whether routine or those resulting from major public health events.

If a health system adapts to minimize the volatility of its outcomes, ensuring reasonable levels of quality and responsiveness, the assessment of its maturity for resilience is proportional to the volatility in the determinants of its behavior. This is a function of its institutional capacity (and/or their aggregation) and varies over time as well.

Rocha et al.’s critique of ‘resilience as disaster response’ (16) parallels our argument that SUS’s chronic instability demands daily resilience practices. For instance, the pre-pandemic volatility in breast cytology tests (Figure 3) reflects a system already operating at its adaptive limits—a finding consistent with their observation that underfunded systems ‘drift into failure’ during routine operations. This reinforces the need to integrate resilience metrics (e.g., variability in EPHF outcomes) into universal health coverage benchmarks to ensure stability amid both routine and crisis demands.

Evaluating the evolution of structural indicators and their volatility over time reveals the inherent variability, which can indicate potential resilience or brittleness, since variability does not necessarily imply negative results (37, 38). However, volatility in essential functions often leads to a mismatch between a system’s capabilities and its demands, creating bottlenecks for interventions in dynamic contexts such as public health. The connection between resilience and performance variability, driven by instability in the foundational elements of health systems, aligns with the margins for resilient performance proposed in Rasmussen’s framework (39). In general terms, this implies that resilience and functional outcomes are not synonymous; maintaining acceptable service levels is also paramount for resilience.

### 4.3 Limitations and further work

While the discussed framework offers a robust conceptual approach to understanding resilience in public health systems through the analysis of service levels and outcome variability, it is not without limitations. One key limitation is its reliance on the availability and quality of known performance indicators over time. In many settings, particularly in low-resource contexts, data on key performance indicators may be incomplete, inconsistent, or unavailable, which could hinder the framework’s practical application. Additionally, the framework assumes that variability (Δv) can be effectively measured and managed, but it may not fully account for the complexity of interactions between multiple determinants of health system performance, such as socio-economic factors, political instability, or cultural influences, which are harder to quantify. Furthermore, while the framework implicitly addresses external shocks through its continuous view of resilience, it does not provide specific guidance on how to prepare for or respond to sudden, large-scale disruptions, such as pandemics or natural disasters. Lastly, the framework’s abstract nature may pose challenges for policymakers and practitioners seeking concrete, actionable steps to operationalize resilience, as it requires a high level of analytical capacity and contextual adaptation.

To overcome the limitations of the framework, efforts should be made to strengthen data collection and management systems, particularly in low-resource settings, to ensure the availability of reliable and consistent performance indicators. This could involve investing in health information systems, training personnel, and leveraging technology for real-time data tracking. Second, the framework could be expanded to incorporate qualitative assessments and contextual factors, such as socio-economic and cultural influences, to provide a more holistic understanding of health system performance and resilience. To address the challenge of external shocks, the framework could be integrated with disaster preparedness and response plans, ensuring that health systems are equipped to handle sudden disruptions. Additionally, translating the framework into practical, actionable guidelines with clear steps and metrics would enhance its usability for policymakers and practitioners. Finally, fostering collaboration between researchers, policymakers, and public health professionals can facilitate the adaptation of the framework to local contexts, ensuring its relevance and effectiveness in diverse settings.

In the same way that different methods can be used to obtain the level of services, the calculation of Dv and its influence on the curvature of α can also be performed by different mathematical models. For example, statistical regression models can be used to demonstrate and predict the correlation between variables, while variance measures and standard deviation can indicate the volatility of these variables over time, as proposed in this study. With this idea, it is possible to define metrics to measure the resilience of the system’s functions based on the variability (volatility) of these indicators over time and prospect their operation using known predictive models, such as applications of Machine Learning, Artificial Intelligence, Fuzzy Logic, etc.

## 5 Conclusion

Resilience in public health is akin to a living theory, constantly evolving and adapting. It thrives on the emergence of new evidence or the challenging of existing beliefs. In the quest to fortify our public health systems, this paper sets the stage for vibrant discussions to come. An opportunity for future investigation lies in longitudinal analyses of EPHFs performance, juxtaposed with recent crises such as the proliferation of arboviruses like Dengue throughout the Americas, as well as outbreaks of diseases like Monkeypox and COVID-19, particularly in Brazil, as outlined in this study, and in other global contexts.

## Data Availability

The original contributions presented in the study are included in the article/supplementary material, further inquiries can be directed to the corresponding author.
